# Ultrasound entropy may be a new non-invasive measure of pre-clinical vascular damage in young hypertensive patients

**DOI:** 10.1186/s12947-015-0006-7

**Published:** 2015-03-20

**Authors:** Caroline Bleakley, Aaron McCann, Vivienne McClenaghan, Paul Kevin Hamilton, Auleen Millar, Richard Pumb, Mark Harbinson, Gary Eugene McVeigh

**Affiliations:** Department of Medical Physics, Queen’s University Belfast, Belfast, Northern Ireland; Department of Cardiovascular Therapeutics & Pharmacology, Queen’s University Belfast, Belfast, Northern Ireland; Department of Cardiology, Belfast City Hospital, Lisburn Road, Belfast BT9 7AB, Belfast, Northern Ireland

**Keywords:** Hypertension, Prevention, Endothelial dysfunction

## Abstract

**Background:**

The identification of pre-clinical microvascular damage in hypertension by non-invasive techniques has proved frustrating for clinicians. This proof of concept study investigated whether entropy, a novel summary measure for characterizing blood velocity waveforms, is altered in participants with hypertension and may therefore be useful in risk stratification.

**Methods:**

Doppler ultrasound waveforms were obtained from the carotid and retrobulbar circulation in 42 participants with uncomplicated grade 1 hypertension (mean systolic/diastolic blood pressure (BP) 142/92 mmHg), and 26 healthy controls (mean systolic/diastolic BP 116/69 mmHg). Mean wavelet entropy was derived from flow-velocity data and compared with traditional haemodynamic measures of microvascular function, namely the resistive and pulsatility indices.

**Results:**

Entropy, was significantly higher in control participants in the central retinal artery (CRA) (differential mean 0.11 (standard error 0.05 cms^−1^), CI 0.009 to 0.219, p 0.017) and ophthalmic artery (0.12 (0.05), CI 0.004 to 0.215, p 0.04). In comparison, the resistive index (0.12 (0.05), CI 0.005 to 0.226, p 0.029) and pulsatility index (0.96 (0.38), CI 0.19 to 1.72, p 0.015) showed significant differences between groups in the CRA alone. Regression analysis indicated that entropy was significantly influenced by age and systolic blood pressure (r values 0.4-0.6). None of the measures were significantly altered in the larger conduit vessel.

**Conclusion:**

This is the first application of entropy to human blood velocity waveform analysis and shows that this new technique has the ability to discriminate health from early hypertensive disease, thereby promoting the early identification of cardiovascular disease in a young hypertensive population.

**Clinical trial registration:**

Clinical Trials.gov, NCT01047423

## Background

As highlighted in the recently released guidelines on the management of hypertension in adults report from the Panel Members Appointed to the Eighth Joint National Committee (JNC 8) [1], in patients with hypertension but no detectable target organ damage, there may be significant subclinical microvascular disease. The ability of the clinician to manage hypertension with the aid of a marker which could detect early microvascular damage would be of enormous value. In hypertension, dysfunction of the microcirculation has been shown to both precede and predict future cardiovascular events and therefore offers an early opportunity to detect disease, acting as a barometer of an individual’s likely future cardiovascular health [2,3]. This study utilized novel spectral analysis techniques to identify changes in Doppler arterial bloodflow waveforms recorded from the carotid and retrobulbar circulation in participants with hypertension compared with a healthy control group.

Wavelet entropy is a measure of waveform complexity, or disorder, derived from the signal’s spectral content [4]. A highly ordered signal’s spectral energy will be concentrated predominantly within one narrow range of frequencies. Conversely, a more disordered signal will result in almost equal distribution of energy content across different frequency bands [4]. The mean wavelet entropy (MWE) of such a signal will be close to zero. Therefore, wavelet entropy succinctly encompasses all the spectral information contained in a blood velocity waveform into a single metric describing the waveform’s complexity. Previous studies have demonstrated a reduction in wavelet entropy, in other words signal complexity, with disease in various biological signals [4-6]. To the authors’ knowledge, this is the first known application of the measurement of entropy to blood velocity waveforms.

## Methods

### Regulatory issues

The study was conducted in line with the Declaration of Helsinki and approved by a local ethics committee. Fully informed written consent was obtained from all participants at study entry.

### Study design

This was a proof of concept study designed to test whether new methods of Doppler ultrasound waveform analysis could distinguish between health and uncomplicated grade 1 hypertension. Hypertensive participants were universally on monotherapy and were switched from their usual therapy to Losartan 100 mg, an angiotensin receptor blocker for 4 weeks prior to study entry in order to in order to eliminate any potential crossover effect from pre-existing therapy. Participants already prescribed Losartan continued as usual.

### Subject characteristics

42 participants with hypertension were recruited together with 26 normotensive control participants. Hypertensive participants meeting the trial entry requirements were screened from the hypertension clinic in Belfast City Hospital. In order to participate in the study, participants had to have grade 1 hypertension (BP >140/90 mmHg) with no evidence of target organ damage detected on fundoscopy, urinalysis or ECG and no other significant medical history. All participants were under 55 years old.

### Procedures

All studies were performed in a quiet temperature regulated room. Participants were asked to refrain from food, drink and smoking from midnight on the day of the study. All participants rested for 5 minutes in the supine position before measures were taken.

### Blood sampling

Using a sterile technique, a butterfly needle was used to draw 10mls of blood into a suitable disposable syringe. Blood was the transferred into a Vaccutainer EDTA tube and a Vaccutainer Serum Clot Activator tube with the tubes labelled with the subject identification number. Samples were then sent via the hospital pod system to Belfast City Hospital Laboratories for processing and analysis.

### Doppler ultrasound and data acquisition

An Esaote MyLab 25™ (Esaote UK, London, UK) ultrasound scanner was used for data acquisition in all participants. Data were converted to a velocity measure automatically via integrated software. Signals from scanners arrive in differing formats, each of which require a customized import module. All measures were obtained automatically using customised software (On-line Vascular Image Analysis v.9.61, London, UK) developed on LabVIEW (National Instruments, Texas, USA). The LabView software produces a text file containing the signal in volts captured from the scanner via the ADC in real time. The single column of data is directly imported.

### Doppler ultrasound of retrobulbar arteries (ophthalmic and central retinal)

**Figure 1:**
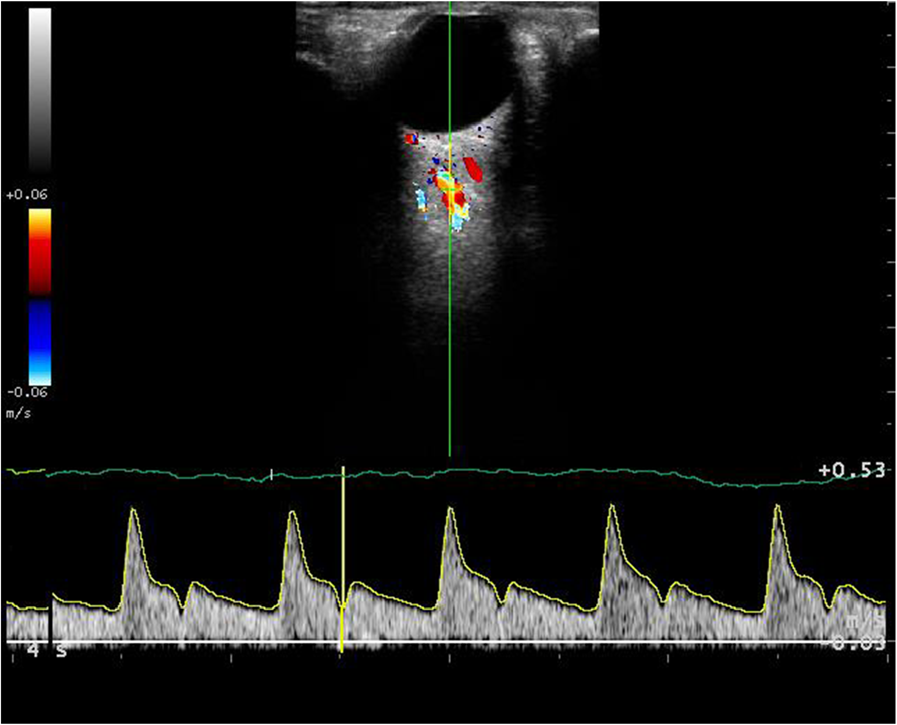
**Doppler ultrasound of right ophthalmic artery.**

Each participant rested in the supine position with the head comfortably supported on one pillow while the right eye was assessed. Involuntary eye movements were minimized by asking the subject to focus the non-examined eye on a fixed point on the ceiling. The transducer was connected to the laptop and the Esaote MyLab Five™ ultrasound scanner. On the Esaote MyLab Five™ ultrasound scanner, the vascular software application was selected, followed by the eyes preset and linear probe with the angle of interrogation at 0^0^. Using colour Doppler, the ophthalmic artery was located along the medial aspect of the optic nerve (Figure 1). An appropriate gate size for the artery was chosen and the pulse repetition frequency (PRF) adjusted so the entire waveform was seen and occupied the largest part of the scale. Minimum and maximum voltages were recorded in the acquisition software. When a stable pulsed Doppler waveform was obtained, 15 to 20 cardiac cycles were recorded. The open recording window on the laptop was then closed and the minimum and maximum voltage values transcribed into the appropriate boxes on the database. The signal was automatically converted to a velocity measure. This process was then repeated for the central retinal artery by locating the artery using colour Doppler as it enters the posterior aspect of the globe in line with the optic nerve.

### Doppler ultrasound of the common carotid artery

Each study participant was positioned supine with the head comfortably supported on one pillow while the right carotid artery was assessed in each patient. On the Esaote MyLab Five™ ultrasound machine the vascular software application was selected, then carotid preset and linear probe. Using colour Doppler, the carotid artery was located along the medial aspect of the jugular vein. The gate size appropriate to the artery was selected and the PRF adjusted so the entire waveform was seen and occupied the largest part of the scale. The minimum and maximum voltages were recorded. When a stable pulsed Doppler waveform was obtained, 15 to 20 cardiac cycles were recorded. The open recording window on the laptop by was closed and the minimum and maximum voltage values were transcribed into the database. The file was converted to a velocity measure automatically via integrated software.

## Signal analysis algorithms

### Summary parameters

#### Time domain analysis

The resistive index (RI) was calculated using:

1 $$ RI=\frac{S-D}{S} $$

where S is the peak systolic velocity and D the end diastolic velocity.

**Figure 2:**
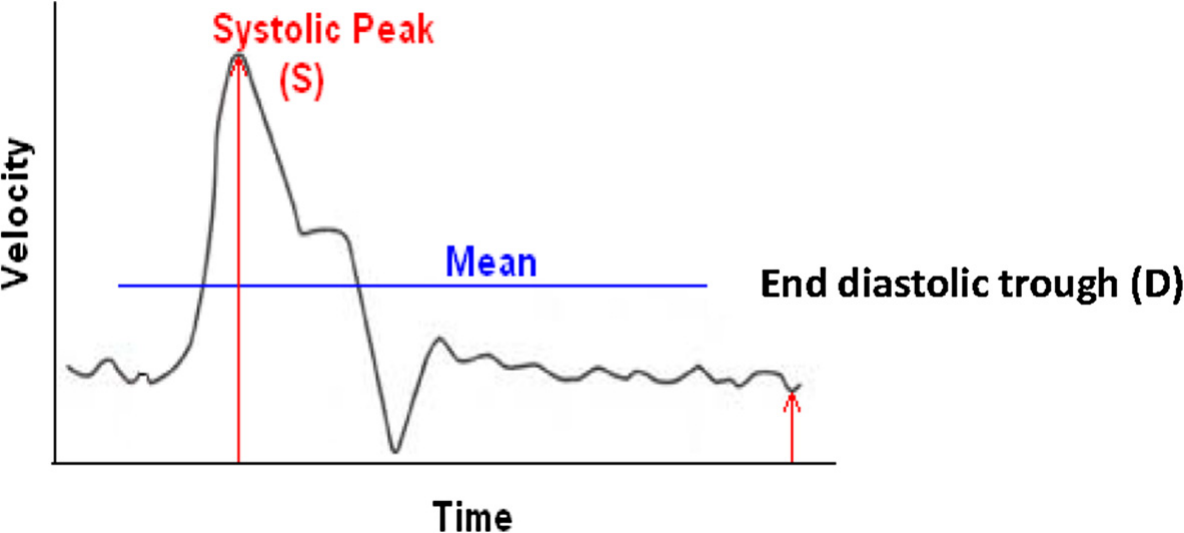
**Calculation of the resistive index and pulsatility index.**

The pulsatility index (PI) was calculated using:

2 $$ PI=\frac{S-D}{M} $$

where S is the peak systolic velocity, D the end diastolic velocity and M the mean velocity [7,8]. These indices were each measured on 3 separate waveforms, with a mean value being used for analysis (Figure 2). Mean velocity was also calculated.

#### Wavelet analysis

Wavelet technology was originally developed in the 1980’s as a means of analysing seismic signals. Morlet et al. [9] were the conceptive group, and since then numerous additive functions have been devised. The wavelet transform allows a window of flexible length to be used; wide to analyse low frequencies and narrow for higher frequencies [10].

**Figure 3:**
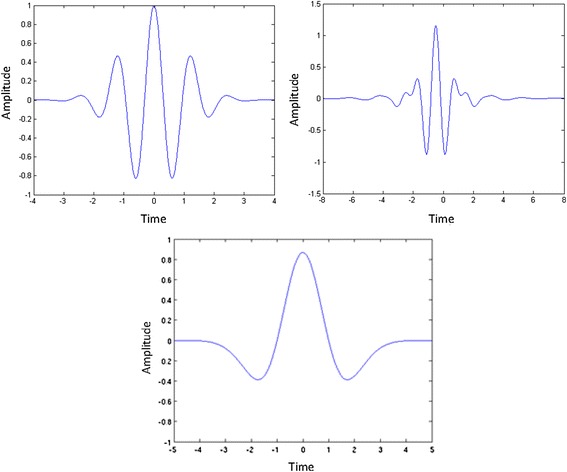
**Examples of wavelets including Morlet, Meyer and Mexican Hat wavelets.**

The discrete wavelet transform (DWT) is a more efficient version than the continuous with respect to programming as it decomposes the signal into components each of which represents a range of frequencies. Wavelets are now available in many different forms, each type more suited to a particular signal than another, allowing the investigator to choose the wavelet most appropriate to the signal they wish to analyse (Figure 3).

The advantage of the wavelet is that it enables localisation of a signal in time as well as frequency therefore allowing analysis of features that vary with time [11]. The time and frequency resolution of wavelet transforms are not fixed, therefore time resolution improves at higher frequencies while frequency resolution increases with lower frequencies [10]. It is therefore most usefully applied to non-time-stationary signals, such as the human blood velocity waveform where it can quantify changes in the entire flow waveform over the whole cardiac cycle [12].

In this study, the DWT analysis of processed blood velocity waveforms was performed using a customised program written in Matlab 2007b (The MathWorks Inc., MA, USA), utilising the Matlab Wavelet Toolbox. A graphical user interface streamlined the analytical process. The ‘Daubechies 8’ wavelet was used for waveforms from all vascular locations. Previous work in our department revealed this to be the most appropriate wavelet for such applications [13]. The output from the wavelet analysis of one signal is considerable. Following DWT analysis, during which frequency data is automatically grouped into 6 bands, the mean signal amplitude in each band calculated. The bands used contain frequencies in the range shown below (Table 1).

**Table 1:** **Frequency bands used in analysis and their constituent frequencies (Herz)**

**Frequency Band**	**Constituent**
	Frequencies (Hz)
2	12.5-25
3	6.25-12.5
4	3.125-6.25
5	1.76-3.125
6	0.87-1.76
7	0.43-0.87

#### Wavelet entropy

**Figure 4:**
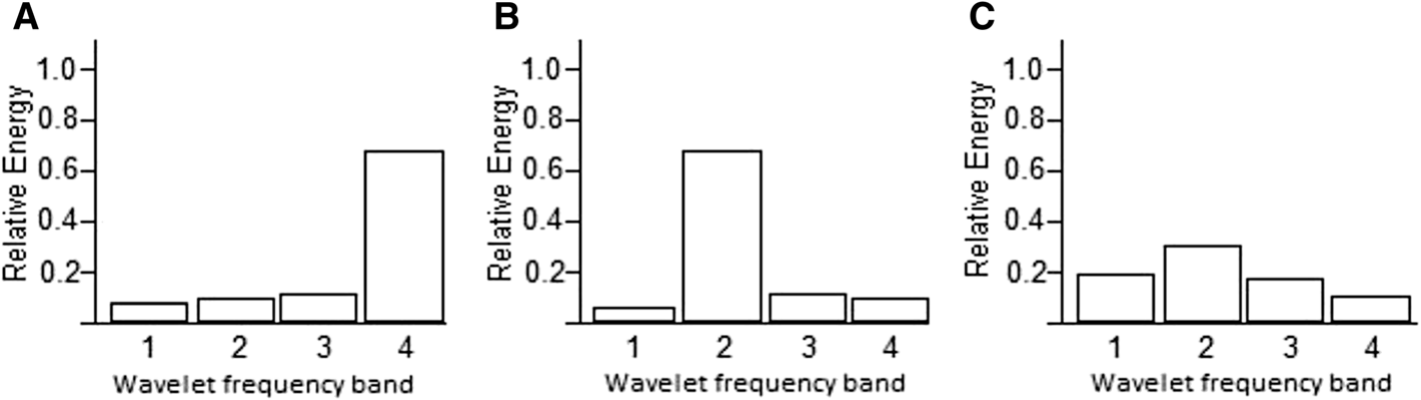
**Wavelet energy distributions corresponding to 4 levels of detail. (A)** An ordered signal with most spectral content within one frequency band. **(B)** An alternative ordered signal with almost all spectral content within one frequency band. **(C)** A disordered signal with energy distribution almostequal in all frequency bands. Adapted from (4).

Wavelet entropy is a measure of waveform complexity, or disorder, derived from the signal’s spectral content [4]. This is represented below (Figure 4) as distribution of energy across individual frequency bands with a highly ordered signal’s spectral energy concentrated predominantly within one frequency band, the relative energy in the frequency band containing the signal will be close to one and close to zero over the remaining frequencies. Conversely, a more disordered signal will result in almost equal distribution of energy content across frequency bands. The mean wavelet entropy (MWE) of such a signal will be close to zero. Therefore, wavelet entropy succinctly encompasses all the spectral information contained in a blood velocity waveform into a single metric describing the waveform’s complexity. Previous studies have demonstrated a reduction in wavelet entropy, in other words signal complexity, with disease [4-6].

#### Mean wavelet entropy

Mean wavelet entropy (MWE) describes the spread of the relative energy spectral distribution as determined by the following equation:

$$ MWE={\displaystyle \sum_m^M\mathrm{R}\mathrm{e}l{E}_m}. In\left(\mathrm{R}\mathrm{e}l{E}_{m.}\right) $$

Where m is the wavelet decomposition level, M is total number of frequency bands and RelE is relative (normalized) energy or amplitude of the signal in each wavelet band. Entropy is unique in that it summarises this spectral composition as a single numeric value and is therefore potentially an extremely attractive marker of vascular function. Measures of entropy have previously been applied in the analysis of vascular dynamics such as heart rate and blood pressure [5,6] and in electroencephalographic (EEG) waveforms [4]. To our knowledge, this method of waveform analysis has not previously been studied in a hypertensive population for assessment of response to therapy or in direct comparison with a healthy cohort.

#### Reproducibility of data

Reproducibility studies were conducted over a period of 10 days on the same healthy volunteer at the same time each day. As is customary, reproducibility was not repeated on the study group. On each day, all measures of vascular function were measured twice by the same operator and reproducibility estimated using a coefficient of variation (Table 2). As studies were performed by a single operator, no inter-observer variability was performed.

**Table 2:** **Reproducibility data for wavelet transform analysis and entropy in carotid, ophthalmic (OA) and central retinal arteries (CRA)**

	**OA**	**CRA**	**Carotid**
	**(CoV)**	**(CoV)**	**(CoV)**
Resistive index	6.17	23.07	4.77
Pulsatility index	20.76	39.11	12.32
Entropy	1.93	2.14	2.14

CoV = coefficient of variation.

### Statistical analysis

Data underwent analysis of variance testing after satisfactory normality of distribution had been demonstrated. Significance was considered to have been reached at the 5% level. Results are presented as means and standard error of the means, and changes from baseline presented as mean (95% Confidence Intervals). Positive results underwent simple linear regression analysis and, if this yielded a significant correlation, these data were then subjected to a multivariate regression model.

## Results

Participants and controls were well-matched in terms of age, sex, heart rate, fasting glucose and renal function (Table 3). There were differences in lipid profiles with respect to total cholesterol (p = 0.05) and triglycerides (p = <0.01), while blood pressure was lower in the control group (systolic 116.6 (standard error of mean 2.44) mmHg vs 142.82 (2.52) mmHg; diastolic 69.00 (1.81) vs 92 (2.3) mmHg; p = <0.001). Smoking status also differed significantly between groups with no control participants currently smoking. None of the control participants were taking any medications while it was expected that hypertensive participants would typically already be taking anti-hypertensive therapy.

**Table 3:** **Baseline demographics of control and hypertension participants**

	**Controls**	**Hypertension**	***p*** **value**
Number	26	42	-
Age (years)	39	45	0.061
Sex (%female)	66.6	69.1	0.488
Body mass index (kgm^−2^)	24.6 (0.7)	28.3 (1.1)	0.013
Weight (kg)	72.6 (2.8)	86.3 (3.3)	0.005
Current cigarette smoking (%)	0	7.3	0.000
Heart rate (beats min^−1^)	62 (1)	64 (0.75)	0.057
Systolic blood pressure (mmHg)	116 (2)	142 (2)	0.000
Diastolic blood pressure (mmHg)	69 (1)	92 (2)	0.000
Fasting glucose (mmol/l)	4.5 (0.211)	5.1 (0.16)	0.062
Total cholesterol (mmol/l)	4.8 (0.80)	5.2 (0.75)	0.045
HDL-cholesterol (mmol/l)	1.6 (0.09)	2.7 (1.26)	0.496
LDL-cholesterol (mmol/l)	2.7 (0.15)	3.0 (0.13)	0.133
Total cholesterol:HDL ratio	3.1 (0.23)	3.9 (0.23)	0.015
Triglyceride (mmol/l)	0.9 (0.12)	1.5 (0.15)	0.005
Proportion with eGFR >60 (l/min/1.73 m^2^)	100	100	1.000

Values are expressed as mean (standard error of the mean). HDL = high density lipoprotein; LDL = low density lipoprotein; eGFR = estimated glomerular filtration rate.

11 participants were already taking an ACE inhibitor, 12 were taking an angiotensin receptor blocker and 5 were taking either a calcium channel blocker or thiazide diuretic. 6 were treatment naïve. Unless already prescribed, all participants were switched to the study drug losartan at the first visit in order to standardize therapy. This was followed by a four week run-in period in order to eliminate any potential crossover effect from pre-existing therapy and to allow participants to be up-titrated to the 100 mg dose of losartan.

In the ophthalmic artery (OA) and central retinal artery (CRA)), entropy was significantly higher in control participants than in those with hypertension (OA = 0.11 (0.05), CI 0.009 to 0.219, p 0.017) (CRA = 0.11 (0.05), CI 0.004 to 0.215, p 0.04) (Table 4).

**Table 4:** **Measures taken from the ophthalmic, central retinal and carotid arteries in control and hypertensive participants**

		**Controls**	**Participants with hypertension**	**Standardised differential mean**	**Confidence interval**	***p*** **value**
Ophthalmic artery	**Entropy**	1.36 (0.03)	1.24 (0.03)	0.12 (0.05)	0.009 to 0.22	0.017
	**Resistive index**	0.88 (0.13)	0.73 (0.01)	0.15 (0.09)	−0.05 to 0.35	0.127
	**Pulsatility index**	1.47 (0.26)	1.15 (0.19)	−0.103 (0.33)	−0.76 to 0.55	0.755
Central Retinal Artery	**Entropy**	1.25 (0.04)	1.14 (0.03)	0.11 (0.05)	0.004 to 0.215	0.040
	**Resistive index**	0.92 (0.04)	0.81 (0.03)	0.12 (0.05)	0.005 to 0.226	0.029
	**Pulsatility index**	2.69 (0.44)	1.73 (0.16)	0.96 (0.38)	0.19 to 1.73	0.015
Carotid Artery	**Resistive index**	0.79 (0.08)	0.78 (0.06)	0.02 (0.11)	−0.20 to 0.23	0.863
	**Pulsatility index**	1.23 (0.42)	1.48 (0.16)	−0.25 (0.37)	−1.01 to 0.49	0.579
	**Entropy**	1.32 (0.02)	1.28 (0.27)	0.04 (0.04)	−0.04 to 0.12	0.267

Results are expressed as standardized differential mean (standard error of the mean) with confidence intervals and corresponding p value for any difference.

For the purposes of being able to compare this new measure with more established methods of vascular assessment, the resistive index and pulsatility index were measured at each arterial site also. Neither of these showed any significant difference between controls and hypertensive participants in measures obtained from the OA. In the CRA, the resistive index (0.12 (0.05), CI 0.005 to 0.226, p 0.029) and pulsatility index (0.96 (0.38), CI 0.19 to 1.72, p 0.015) were higher in controls than hypertensive participants (Table 4). No significant differences were seen in the carotid artery in any of the measures obtained. No significant correlation was demonstrated between RI, PI and entropy (RI and entropy p = 0.075, PI and entropy 0.012 using Pearson’s correlation coefficient).

As the main endpoint of the study, entropy underwent further analysis in order to extract any possible relationship between it and baseline characteristics. In the first instance simple linear regression was applied (Table 5) and, if this yielded a significant correlation, these data were then subjected to a multivariate regression model (Table 6). The results of this analysis of the entropy data indicate that this measure is significantly influenced by age and systolic blood pressure (r values 0-4-0.6). Other baseline characteristics that may have been presumed to influence entropy, such as diastolic blood pressure and total cholesterol, in fact did not show a significant association. For this reason multivariate regression was restricted to the analysis of age and systolic blood pressure for their effect on entropy. After such analysis, a plausible correlation still exists indicating that entropy is to some extent dependent on these factors and may therefore be a potentially robust measure of vascular health.

**Table 5:** **Linear regression analysis of entropy**

	**r value (p value) OA**	**r value (p value) CRA**
Systolic BP (mmHg)	−0.46 (<0.001)	−0.34 (0.02)
Diastolic BP (mmHg)	−0.15 (0.29)	0.003 (0.98)
Age (years)	−0.51 (<0.001)	−0.19 (0.18)
Total cholesterol (mmol/l)	−0.13 (0.35)	−0.03 (0.84)

Results are given as the r value with the corresponding p value for significance. OA = ophthalmic artery, CRA = central retinal artery.

**Table 6:** **Multiple regression of age and systolic blood pressure (corrected for age, systolic blood pressure, diastolic blood pressure and total cholesterol)**

	**r value (p value) OA**	**r value (p value) OA**
Systolic BP (mmHg)	−0.27 (0.12)	−0.28 (0.13)
Age (years)	−0.48 (0.01)	−0.28 (0.14)

Results are given as the r value with the corresponding p value for significance. OA = ophthalmic artery, CRA = central retinal artery.

## Discussion

A wealth of evidence exists to support the link between hypertension and cardiovascular risk and yet there is concern that younger individuals are inadequately served by short-term risk prediction. As a consequence, the assessment of overall lifetime risk of a cardiovascular event is gaining credence [14]. In order to more accurately risk stratify individuals, there is a need to identify reliable surrogate predictors of cardiovascular disease that would detect early changes in the vasculature which to date have evaded clinicians.One means of detecting these small changes is by the use of Doppler ultrasound. This study examined the use of entropy, a novel summary measure of Doppler ultrasound waveform analysis, for the first time in a young hypertensive cohort.

Traditionally, the detection of such changes in blood flow signals relied upon time domain descriptors such as the resistive (RI) and pulsatility (PI) indices. They were included as comparators in this study as they are widely quoted and understood, being often displayed directly on ultrasound scanner screens now. These measures have been used extensively in the analysis of Doppler blood flow waveforms and yet their limitations have been equally well documented [12,15-19]. As single domain methods, it has been suggested that they lack the necessary flexibility to adequately assess an entity as variable as the blood flow waveform. Derived from single inflection points during the cardiac cycle, they possess limited ability to account for the ebb and flow of the cycle. This may be a reason for the lack of correlation seen between these techniques and entropy in this study.

In this study, lower RI and PI values were seen in the hypertensive cohort in the central retinal artery (CRA) but no significant difference was demonstrated in the ophthalmic artery (OA). This non-conformity of results with the use of these measures is not unexpected. These indices have been questioned as to whether they are suitably reliable to inform conclusions with varying degrees of performance in the literature [15-17, 20]. Of added concern, there is non-uniformity of evidence as to the direction in which the RI should be affected in hypertension, with some studies reporting an increase in this measure while others report a reduction. The reason for this discrepancy perhaps lies in what the RI is actually measuring. If taken literally, it should be viewed as a straightforward measure of resistance, however, this may be a misnomer as the RI is actually dependent on the interaction between resistance and compliance which together comprise the arterial waveform [17]. For instance, it is expected that those with hypertension will have reduced vascular compliance compared with non-hypertensive counterparts, which will result in a lower RI in these individuals even if the level of vascular resistance is comparable to that of the control participants [17]. In other words, if compliance was to differ between participants then the RI may change even though the vascular resistance is the same.

An alternative method of analysis such as the short-time Fourier transform would have been subject to significant limitations in the setting of a study looking at blood velocity waveforms. Time domain descriptors of the flow velocity waveforms are limited to isolated points of the cardiac cycle. The short-time Fourier transform (STFT) deconstructs the signal into its frequency components and the position in time at which these frequencies are observed. It allows time-frequency representation of the flow velocity envelope, while still requiring stationarity of the signal within a finite time interval [10]. In practice, a 10 millisecond window is frequently chosen as an assumed interval over which the signal displays stationarity [10]. As a result of this fixed time-frequency window, the STFT is of limited use in the analysis of signals with wide bandwiths that change rapidly over time [10]. With respect to its application to blood velocity waveform analysis, the STFT is certainly restricted by the Fourier requirements for signal time-stationarity and linearity of the system within the chosen window of analysis.

With the limitations of these existing techniques in mind, this study focussed on the novel use of entropy, which has never before been applied to the analysis of biological waveforms. Wavelet entropy is a measure of waveform complexity, or disorder, derived from the signal’s spectral content [4]. A highly ordered signal’s spectral energy will be concentrated predominantly within one frequency band, while a more disordered signal results in almost equal distribution of energy content across frequency bands. Entropy is unique in that it summarises this spectral composition as a single numeric value and is therefore potentially an extremely attractive marker of vascular function.

This study investigated whether this measure could detect differences in the microvasculature between a hypertensive and healthy cohort. Significantly lower entropy values were seen in the ophthalmic and central retinal arteries in the hypertensive cohort with no significant change seen in the larger conduit vessel. In simplified terms, the higher the value of entropy the greater the degree of disorder or complexity. The findings in the present study of a decrease in wavelet entropy, or signal complexity, in the hypertensive cohort are in keeping with previous studies which have demonstrated a reduction in signal complexity in other disease states, although not involving the application of the technique to a blood velocity envelope [4-6]. This result suggests that entropy could be used in future models to provide a quantitative analysis of microvascular healthas it provides a single metric which is easily appreciated by clinicians.

However, the result is difficult to interpret as this is the first known application of this novel method of spectral analysis to a blood velocity envelope in the assessment of early cardiovascular disease. What is interesting though is that the measurement of entropy was only significant in the smaller distal vessels rather than in the conduit system. This finding is more in keeping with what should be expected from an assessment of these vessels, with the larger feeding artery lying too proximally in order to accurately show changes in the microvasculature which should be more readily seen in distal vessels which abut the microcirculation. As this study allowed direct comparisons to be made between methods of ultrasound interrogation, this could mean that entropy is actually a more sensitive and specific measure of microvascular function than either of the more established means it was compared with.

The study is of course subject to the usual caveats of a small sample size which may have resulted in either an over or underestimation of any differences detected. It must also be noted that the RI and PI values in this study were gained from the analysis of 3 separate waveforms while the entropy analysis was determined from 15–20 waveforms. This difference is potentially a limitation in the study analysis. The issue of confounding is also an important one, as there were certainly differences detected between the controls and those with hypertension with respect to baseline characteristics. In the main, these differences were as expected between two such groups in variables such as systolic and diastolic blood pressure along with lipid levels which although would not be considered abnormally high, were still higher in hypertensive participants. Although it is possible that these differences had minor effects on outcomes, it is unlikely that they had a major impact as the participants recruited into each group are largely representative of two such populations. A larger study would certainly be required to more fully interrogate this development in the detection of early preclinical microvascular disease.

Despite these caveats entropy is a novel and innovative technique that has been used in this study expressly for the purpose of tracking changes within microvascular function that existing Doppler ultrasound methods have been inadequately sensitive to detect. It seems plausible that the changes detected in this study signify the earliest evidence of microvascular dysfunction which previously has been beyond the sensitivity of non-invasive detection. This is the first description of microvascular dysfunction in a young and otherwise healthy hypertensive cohort that has been detected by such novel means.

## Conclusion

While this measure is undoubtedly in its infancy in relation to the early detection of cardiovascular disease, these initial results suggest that entropy has the ability to discriminate health from early disease. This is important, as these changes were not always obvious with traditional measures, and therefore this could represent an opportunity to identify previously veiled microvascular alterations. As a marker of microvascular dysfunction, entropy is very attractive as it offers a single metric that could be easily interpreted by clinicians seeking to identify those with early microvascular damage despite favourable risk factor profiles. In conclusion therefore, this study has presented the potential for a novel method of waveform interrogation to be used in the early identification of cardiovascular disease in order to aid risk stratification in a young hypertensive population.

## Abbreviations

CRA: Central retinal artery
OA: Ophthalmic artery
MWE: Mean wavelet entropy
RI: Resistive index
PI: Pulsatility index
DWT: Discrete wavelet transform
EEG: Electroencephalographic
STFT: Short-time fourier transform
